# A case report on concurrent occurrence of systemic mastocytosis and myeloid sarcoma presenting with extensive skin involvements and the results of genetic study

**DOI:** 10.1097/MD.0000000000021948

**Published:** 2020-12-11

**Authors:** Xinye Wang, Lu Zhang, Daobin Zhou, Hao Cai, Xuan Wang, Xianyong Jiang

**Affiliations:** Department of Hematology, Peking Union Medical College Hospital, Chinese Academy of Medical Sciences, Beijing, China.

**Keywords:** Arid1a, Kit, myeloid sarcoma, systemic mastocytosis

## Abstract

**Introduction:**

Systemic mastocytosis is a rare disease due to mast cell accumulation in various extracutaneous sites. Systemic mastocytosis with an associated clonal hematologic non-MC lineage disease is the second most common subtype of systemic mastocytosis. The most common mutation associated with both systemic mastocytosis and myeloid sarcoma is mutation in *Kit*. Here, we identified the novel KIT D816V and ARID1A G1254S mutations co-occurring in systemic mastocytosis with myeloid sarcoma.

**Patient Concerns:**

A 33-year old male patient presented multiple skin lesions for 10 years. Symptoms accelerated in 2017 with decreased body weight. Physical examination revealed enlarged lymph nodes in his neck, axilla and inguinal region; conjunctival hemorrhage; gingival hyperplasia. Skin biopsy showed mast cell infiltration. Flow cytometry detected CD2, CD25 and CD117 positive cells in lymph nodes. Codon 816 KIT mutation D816V and codon 1245 ARID1A mutation G1254S were found in peripheral blood. MPO, CD117, CD68 positive cells in lymph nodes indicated co-existing myeloid sarcoma.

**Diagnosis:**

Systemic mastocytosis with an associated clonal hematologic non-MC lineage disease of myeloid sarcoma

**Interventions:**

Cytarabine and daunorubicin for myeloid sarcoma and dasatinib for systemic mastocytosis were initiated. Anti-histamine and anti-leukotrienes therapy were used to prevent NSAIDs-induced shock. Platelets were infused to treat bone marrow suppression.

**Outcomes:**

Patient was discharged after recovered from bone marrow suppression. Dasatinib continued on outpatient.

**Conclusion:**

This is the first case of patient with systemic mastocytosis and myeloid sarcoma simultaneously presenting extensive skin involvements. Mutations of *Kit* and *Arid1a* emphasis the importance to notice possibility of various tumors occurring in patients with multiple mutations. In addition, cysteine-leukotrienes-receptor antagonists should always be used to prevent anaphylactic shock due to mast cell activation.

## 1 Introduction

Mastocytosis is a rare disorder due to mast cell accumulation in multiple sites. It is divided into 2 groups: cutaneous mastocytosis and systemic mastocytosis (SM). While cutaneous mastocytosis only involves the skin, SM can invade at least 1 extracutaneous organ. According to 2008 World Health Organization diagnosis criteria,^[1]^ to diagnose SM requires either the major criteria of multifocal dense infiltrations of mast cells in extracutaneous organs (more than 15 mast cells aggregating) with 1 of 4 minor criteria (more than 25% mast cells in extracutaneous organs show an abnormal morphology; codon 816 KIT mutation D816V detected in extracutaneous organs; mast cells in bone marrow express CD2, CD25 or both; more than 20 ng/mL serum tryptase) or 3 minor criteria. Once diagnosed SM, subtypes of SM need to be identified according to organ invasions and presence of other types of tumors. Subtypes of SM include^[2]^: indolent systemic mastocytosis characterized by a stable and slowly progressing clinical course; systemic mastocytosis with an associated clonal hematologic non-MC lineage disease (SM-AHNMD); aggressive systemic mastocytosis defined as SM resulting tissue dysfunction such as hepatic fibrosis; mast cell leukemia that have more than 10% immature mast cells in peripheral blood or more than 20% immature mast cells in bone marrow; the last 2 types are mast cell sarcoma and extracutaneous mastocytoma. SM-AHNMD is the second most common variants among these subtypes of SM, whose prognosis depends on the nature of associated tumors.^[3]^

Myeloid sarcoma consists of myeloid blasts that occur outside bone marrow. It can precede or coincide with acute myeloid leukemia (AML).^[4]^ Detection of myeloid sarcoma is equivalent to the diagnosis of AML.^[5]^ The immunohistochemistry is used to confirm the diagnosis: the most common markers are MPO, CD117, and CD68.^[6]^ Multiple mutations have been reported to be linked with myeloid sarcoma. Cytogenetic studies have shown that myeloid sarcoma can carry the same aberrations as those seen in AML, such as *Npm1*, *Flt3, Cebpa*, *MLL* rearrangement, inv (16), and the most common t (8;21). Other mutations in *Kit*, *Idh2*, and *Braf* genes were also identified in myeloid sarcoma.^[7]^

Herein we reported a case of SM co-occurring with myeloid myeloma whose genetic profiles showed a cooccurrence of KIT D816V and ARID1A G1254S mutations. This case, according to our limited knowledge, was also the first case of patient with SM and myeloid sarcoma presenting as extensive skin involvements.

## 2 Case report

Patient has provided informed consent for publication of the case. A 33-year old male patient presented diffuse brownish skin lesions on his trunk and extremities in 2004. He is a constructor and has been exposed to toxic chemicals. Before hospitalization, the skin biopsy showed massive infiltration of eosinophils. In January 2017, the patient complained drop of body weight (10 kg in 2 years), fusion, and enlargement of his rashes. Physical examination revealed multiple enlarged lymph nodes. Lab tests showed elevated white blood cells (93.1 × 10^9^/L, unclassified), abnormal liver functions (glutamyl transpeptidase 195U/L, alkaline phosphatase 247 U/L, serum lactate dehydrogenase 374 U/L). Blood smear revealed 19% promyelocytes and 24% metamyelocytes of white blood cells. Computer tomography showed hepatomegaly, uneven density in spleen, and multiple enlarged lymph nodes in retroperitoneum. Lymph node biopsy suppurative lymphadetitis. Bone marrow aspiration resulted in dry tap and bone marrow biopsy found no abnormalities except for active proliferation. No abnormalities were found through bone marrow chromosome karyotyping. Mutation screening tests from peripheral blood revealed codon 816 KIT mutation D816V and codon 1245 ARID1A mutation G1254S. The family history is non-significant. Treatment of hydroxyurea was not effective. The use of imatinib was discontinued due to his personal reasons.

**Figure 1:**
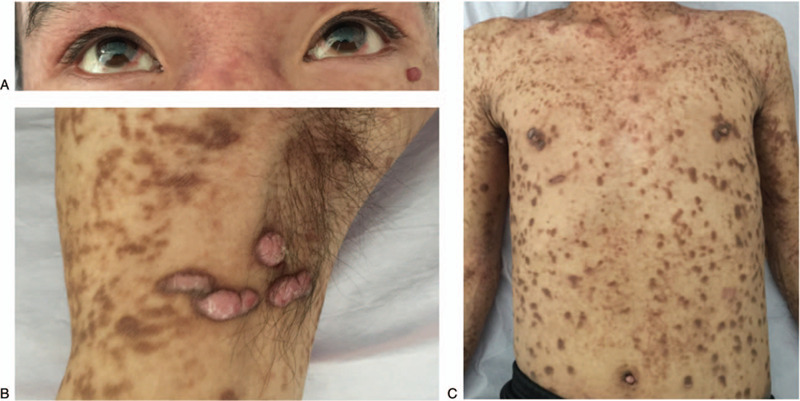
Multiple cutaneous lesions, conjunctivitis and enlarged lymph nodes in patients. Physical examinations in 2017 confirmed bilateral conjunctival hemorrhage (A), enlarged lymph nodes in his right axilla (B) and multiple skin lesions with hyperpigmentation on the trunk and extremities (B and C).

On admission (May 2018), physical examinations confirmed multiple skin lesions with hyperpigmentation; enlarged lymph nodes in his neck, axilla and inguinal region; conjunctival hemorrhage; gingival hyperplasia (Fig. 1). Screening tests for infection were negative. Bone scintigraphy detected elevated metabolism over his body. With eosinophil infiltration in skin biopsy and *Kit* mutation, systemic mastocytosis was suspected. To further confirm the diagnosis, biopsy was performed in the patient's skin (axilla), lymph nodes (right inguinal region), and bone marrow, as well as the flow cytometry and Sanger sequencing. Massive staining with toluidine blue in skin tissue and lymph nodes indicated mastocytosis. Immunohistochemical staining of skin tissue was positive for CD117, CD68, CD30, CD3, CD21, CD5, CD34, BF-1, and the Ki-67 labeling index was 10%; the cells were negative for MPO, S-100, CD1a, CD20. Flow cytometry of lymph node tissue in right inguinal region detected that 13% cells were CD2, CD25, and CD117 positive, CD4, CD56, and CD123 negative. Sanger sequencing showed heterozygous mutation of *Kit*. Lymph nodes in right inguinal tissue which were positive for MPO, CD117, CD68, CD30, Ki-67 (labeling index 80%), PAX-5, CD21, CD2 and TdT, negative for CD1a, AE1/AE3, and S-100 in immunohistochemical staining indicated co-occuring myeloid sarcoma. According to the World Health Organization criteria,^[1]^ the diagnosis of systemic mastocytosis with an associated clonal hematologic non-MC lineage disease (SM-AHNMD) was confirmed.

Chemotherapy (cytarabine 0.15 g, q12 h, sc, day1–7; daunorubicin 95 mg, qd, iv, day 1–3) for myeloid sarcoma and dasatinib (100 mg, qd, po) for systemic mastocytosis were initiated simultaneously (Day 1, July, 2018). Posaconazole was used to prevent fungi infection. Patient was monitored by electrocardiogram. On day 2, patient had diarrhea and fever after unclean diet. Broad spectrum antibiotics and nonsteroidal antiinflammatory drugs (NSAIDS) were initiated. After 30 minutes of talking NSAIDs, the patient experienced dyspnea, tachycardia. Blood pressure dropped to 70/30 mm Hg. Distributive shock was suspected. Norepinephrine and fluid therapy could reverse the situation. Considering vulnerable condition of the object, chemotherapy was discontinued. On day 3, the patient was febrile again. After the use of NSAIDs, patient had shock attack again. Distributive shock due to degranulation of mast cells was suspected. NSAIDs treatment was stopped. Anti-histamine and anti-leukotrienes therapy were initiated and shock did not occur thereafter. On day 13 of the therapy, bone marrow suppression (platelet 20 × 10^9^/L, white blood cells 2.96 × 10^9^/L, neutrophils 1.84  × 10^9^/L, hemoglobulin 103 g/L) occurred. 1-unit platelets were infused. On day 15, broad spectrum antibiotics were switched to cefaclor as diarrhea was relieved. On day 21, the number of platelets and white blood cells recovered. Patient was discharged. Dasatinib continued on outpatient.

## 3 Discussion

The patient exhibited multiple skin lesions with enlarged lymph nodes. Biopsy in multiple sites and codon 816 KIT mutation D816V confirmed the diagnosis of SM-AHNMD. This is the first case of patient with systemic mastocytosis and myeloid sarcoma presenting as extensive skin involvements. There was only 1 report of systemic mastocytosis with myeloid sarcoma and the patient presented compressive myelopathy.^[8]^ No genetic analysis was done in previous case. In this case, the patient carried heterozygous *Kit* mutation which was known to be related to mastocytosis. KIT protein is a tyrosine kinase receptor that is responsible for activating phosphorylation cascade in various transcription factors.^[9]^ Researches have shown KIT D816V mutation, which was identified in more than 80% of patients with SM, induced autophosphorylation of KIT receptor.^[10]^ In addition, the mutation of AT-rich interaction domain 1A (*Arid1a*) was found in the patient. ARID1A is a member of SWItch/sucrose Non-fermenting family and is responsible for regulating transcription of various genes by changing chromatin structures.^[11]^ Evidences have identified *Arid1a* as a tumor suppressor gene,^[12]^ functioning in cell cycle regulation. The mutation of *Arid1a* was associated with a broad spectrum of cancers, such as lung adenocarcinoma,^[13]^ ovarian cancer,^[14]^ and gastric cancers.^[15]^ Interestingly, mutations of *Kit* and *Arid1a* have been found in various types of tumors. Phenotypical analysis in a group of endometriosis-associated ovarian cancers revealed aberrant expression of KIT and downregulation of ARID1A.^[16]^ Therefore, it is likely to assume that both *Arid1a* and *Kit* mutations contribute to myeloid sarcoma co-occurring mastocytosis in our patient. Indeed, a whole-exome sequencing analysis detected both *Kit* and *Arid1a* mutations in 69 hepatocellular carcinoma samples. As it demonstrated that *Kit* mutated in a fully clonal manner and *Arid1a* mutated heterogeneously, *Kit* mutation may precede *Arid1a* mutation.^[17]^ Moreover, while *Kit* mutated universally in gastrointestinal stromal tumors (GIST), only a portion of GIST harbored mutations of *Arid1a*,^[18]^ indicating ARID1A may act as a downstream protein of KIT-related pathways. However, no molecular studies provided direct evidence of KIT interacting with ARID1A. Further researches are needed to reveal the mechanism. Since mutations of *Kit* and *Arid1a* coexisted in various cancers, it is critical to notice if there could be any signs of other tumorigenesis in our patient during follow-up therapy.

The therapeutic strategy for SM-AHNMD was adopted as general recommendation^[1]^: treat hematological malignancy and mastocytosis independently. Therefore, our patient was treated with chemotherapy for myeloid sarcoma and dasatinib for mastocytosis simultaneously. Researches have demonstrated KIT mutation D816V as one of the most common resistance to imatinib and sunitinib,^[19]^ while dasatinib has been shown to overcome the resistance.^[20]^ In a clinical trial involving 50 patients with advanced GIST, dasatinib was proven to increase 6-month progression-free survival by 30%.^[21]^ Therefore, we chose dasatinib to treat systemic mastocytosis in this case.

The patient exhibited shock on day 2 of the therapy. No abnormalities in electrocardiogram and myocardial enzyme panels ruled out the possibility of cardiogenic shock. The level of D-dimers did not elevate. The ultrasound for lower extremities yielded negative findings. Therefore, obstructive shock was less likely to occur. History of ingesting unclean food and symptoms of diarrhea and fever directed to the diagnosis of distributive shock due to infection. However, broad spectrum antibiotics did not prevent the second shock on day 3 of the therapy, indicating shock due to other etiologies. It was noticed that the patient had shock attack every time after use of NSAIDs, which was reported to trigger mast cell activation.^[22]^ Cysteinyl leukotrienes and platelet-acting factors released by mast cells can increase vascular permeability and vasodilation,^[23,24]^ thus causing severe anaphylaxis in patients.^[25]^ Strategies to cope with NSAIDs induced anaphylactic reaction include intramascular epinephrine and intravenous diphenhydramine or glucocorticoids during acute anaphylaxis, daily histamine receptor antagonists, daily serotonin receptor antagonists, and cysteine-leucotriene-receptor anatagonists.^[2]^ Therefore, NSAIDs were avoided in the patient. After our patient was given montelukast and cetirizine, anaphylactic shock never appeared.

## 4 Conclusion

We first report a patient presenting as extensive skin involvements with systemic mastocytosis and myeloid sarcoma in same time. Mutations of KIT D816V and ARID1A G1254S both contributed to carcinogenesis, which highlighted the power of genetic analysis in patients with SM-AHNMD. Physicians should be careful about onsets of various tumors in SM-AHNMD patients with mutations of multiple tumor suppressor genes. In addition, cysteine-leukotrienes-receptor antagonists should be used to prevent anaphylactic shock due to mast cell activation during the treatment of systemic mastocytosis.

## Author contributions

Dr. Xinye Wang is responsible for manuscript writing and resoning. Dr. Lu Zhang is responsible for the support of the study and revision of the manuscript. Dr. Daobin Zhou, Dr. Hao Cai, Dr. Xuan Wang and Dr. Xiangyong Jiang are responsible for patient management.
